# A Cost-Effectiveness Analysis of Systemic Therapy for Metastatic Hormone-Sensitive Prostate Cancer

**DOI:** 10.3389/fonc.2021.627083

**Published:** 2021-02-24

**Authors:** Winnie W. Y. Sung, Horace C. W. Choi, Peter H. Y. Luk, Tsz Him So

**Affiliations:** ^1^ Clinical Excellence Research Center, Stanford University School of Medicine, Stanford, CA, United States; ^2^ Department of Clinical Oncology, Queen Elizabeth Hospital, Hong Kong, Hong Kong; ^3^ Department of Clinical Oncology, University of Hong Kong, Hong Kong, Hong Kong

**Keywords:** prostate cancer, abiraterone (AA), enzalutamide (ENZ), apalutamide, docetaxel, cost effectiveness

## Abstract

**Background:**

Currently, approved first-line treatment options of metastatic hormone-sensitive prostate cancer (mHSPC) include (1) androgen deprivation therapy (ADT) alone, ADT plus one of the following: (2) docetaxel, (3) abiraterone, (4) enzalutamide, and (5) apalutamide. The high cost of novel androgen receptor pathway inhibitors warrants an understanding of the combinations’ value by considering both efficacy and cost.

**Objective:**

This study aimed to compare the cost-effectiveness of these five treatment options in mHSPC from the US payer perspective to guide treatment sequence.

**Methods:**

A Markov model was developed to compare the lifetime cost and effectiveness of these five first-line treatment options for mHSPC using outcomes data from published literature. Health outcomes were measured in life-years and quality-adjusted life-years (QALYs). Drug costs were obtained from the Veterans Affairs Pharmaceutical Catalog. We extrapolated survival beyond closure of the trials.

**Outcome Measurements and Statistical Analysis:**

Life-years, QALYs, lifetime costs, and incremental cost-effectiveness ratios (ICERs) were estimated. Univariable, 2-way, and probabilistic sensitivity analyses were performed to evaluate parameter uncertainty. A willingness-to-pay (WTP) threshold of US$100,000 per QALY was used.

**Results:**

Compared to ADT alone, docetaxel plus ADT provided a 0.28 QALY gain at an ICER of US$12,870 per QALY. Abiraterone plus ADT provided an additional 1.70 QALYs against docetaxel plus ADT, with an ICER of US$38,897 per QALY. Compared to abiraterone plus ADT, enzalutamide plus ADT provided an additional 0.87 QALYs at an ICER of US$509,813 per QALY. Apalutamide plus ADT was strongly dominated by enzalutamide plus ADT. Given the WTP threshold of US$100,000 per QALY, abiraterone plus ADT represented high-value health care.

**Conclusions:**

Abiraterone plus ADT is the preferred treatment option for men with mHSPC at a WTP threshold of US$100,000 per QALY.

## Introduction

In the US, prostate cancer accounts for one in five new cancers, making it the most diagnosed cancer in men; it is also the second most common cancer-related death in men (1). Despite a declining incidence of prostate cancer, the incidence of metastatic prostate cancer is increasing (2). Metastatic prostate cancer has a 5-year overall survival rate of 30.5% (3). The treatment landscape for metastatic hormone-sensitive prostate cancer (mHSPC) has significantly changed over the past decade, with emerging evidence supporting the addition of novel agents including chemotherapy or androgen receptor pathway inhibitors to the backbone treatment of androgen deprivation therapy (ADT).

In 2015, STAMPEDE (4) and CHAARTED (5) were the two pivotal clinical trials that demonstrated an overall survival (OS) benefit of upfront docetaxel in addition to ADT in patients with mHSPC. In 2017, LATITUDE (6), and STAMPEDE (7) trials showed a similar degree of OS benefit of upfront abiraterone in addition to ADT in this group of patients. More recently, ENZAMET (8) and ARCHES (9) trials demonstrated an OS benefit of upfront enzalutamide plus ADT; and TITAN (10) trial similarly demonstrated OS benefit of upfront apalutamide plus ADT.

There is no head-to-head comparison of different agents and a randomized controlled trial comparing all agents is unlikely feasible. The emergence of these treatment options in the castration-naïve setting has led to new challenges in finding the best treatment sequence for mHSPC. Network meta-analyses reported so far offered no clear answer. Sathianathen et al. suggested that different combination strategies were statistically comparable with none being clearly superior (11).

Our group previously reported that abiraterone appears to be more effective than other treatment options in reducing risk of death and preventing disease progression (12).

With an aging population and improved survival outcomes, mHSPC represents an increasing economic burden to healthcare systems in developed countries. Previous cost-effectiveness analysis comparing ADT alone, ADT in combination with docetaxel or abiraterone from a US private payer perspective demonstrated that docetaxel plus ADT was cost-effective with an incremental cost-effectiveness ratio (ICER) of US$34,723, compared to abiraterone plus ADT with an ICER of US$295,212 (13). With the entrance of enzalutamide and apalutamide as new treatment alternatives and reports of longer follow up data available, there is a need to compare the cost-effectiveness of currently approved treatment regimens in mHSPC to guide management.

## Patients and Methods

### Model Overview

A Markov state-transition model was developed to evaluate the costs and health outcomes of treating mHSPC with one of the following: (1) ADT alone; (2) docetaxel plus ADT; (3) abiraterone plus ADT; (4) enzalutamide plus ADT; or (5) apalutamide plus ADT. ADT alone was the referent strategy, as it was the control arm across mHSPC trials. The model considered a hypothetical cohort of 70 kg 60-year-old men with newly-diagnosed mHSPC transitioning through three discrete health states: progression free, progression, and death (**Supplementary Figure 1**). All mHSPC patients started with progression free and either remained at that state or transitioned to progression to castration resistant prostate cancer (CRPC) or death. Once in progression, patients could remain in that state or transition to death (**Supplementary Figures 2a, 2b**). We did not include a separate health state for symptomatic disease because it was assumed most patients with mHSPC have certain degree of symptoms and clinical progression to CRPC was included as part of the definition for “progression” in reported trials.

Primary model outputs included cost, life-years (LYs), and quality-adjusted life years (QALYs), which were used to calculate the ICERs. Costs were calculated from the US health payer perspective. Costs, LYs and QALYs were accumulated over a lifetime horizon at one-month cycles and discounted at 3% per year. Willingness-to-pay (WTP) threshold of US$100,000 was used (14). Model calibration was conducted with R software (version 3.6.1); model development and statistical analysis were performed with Amua software (15).

### Model Survival and Progression Risk Estimates

The transition probability of progression-free to CRPC for each treatment strategy was estimated based on the progression-free survival (PFS) curves in respective clinical trials (**Supplementary Figure 3**). We considered exponential, Weibull, and log-normal distributions when fitting the published PFS curves. A log-normal distribution was chosen as it provided the best fit among other distributions according to the Akaike information criterion (AIC). We assumed a single, time-independent transition probability from CRPC to death for all strategies and estimated this probability by calibrating to the published overall survival (OS) curves (**Supplementary Figure 3**). The US life tables were used to estimate the risk of all-cause mortality (**Supplementary Table 2**).

### Treatment Strategies

Docetaxel was administered at 75 mg/m^2^ every 3 weeks for six cycles. Abiraterone was administered at a dose of 1,000 mg daily, enzalutamide at 160 mg daily, and apalutamide at 240 mg daily until progression. For all strategies, ADT was administered until progression. We included grade 3 or above adverse events which had significantly different rates between treatments and occurred in at least 1% of patients in published literature (**Supplementary Table 1**). We assumed that adherence to treatment was 100%.

### Costs

Only direct medical costs were considered, including drug, administration, and adverse event costs. Costs for ADT, docetaxel, abiraterone, enzalutamide, and apalutamide were estimated using prices listed in the Veterans Affairs (VA) Pharmaceutical Catalog (16). The costs were estimated primarily from a health care sector perspective. The cost of treatment for CRPC patients was derived from published literature (13). Costs of prostate-related death included in-patient care, hospice care, emergency room visits, office visits, and outpatient procedures (17). The costs for treatment related toxicities are outlined in **Supplementary Table 1**. All costs were converted to a one-month cycle and inflated to 2020 US dollars using the US consumer price index.

### Utilities

QALYs are estimated by adjusting survival time with health-related quality of life. Health utilities for patients on ADT, abiraterone and docetaxel are derived from published literature (13, 18–20). The disutility of treatment arising from docetaxel was only applied during the six cycles when the treatment was received, and then the utility for metastatic patients on ADT was applied. The health utilities for patients on enzalutamide and apalutamide were assumed to be the same as patients on abiraterone, based on the reported trend of delayed time to pain progression and fatigue and pain severity (21, 22). The utilities of abiraterone, enzalutamide, apalutamide, and ADT were applied throughout the duration of treatment. The disutilities for treatment-related adverse events are outlined in **Supplementary Table 1**.

### Sensitivity Analysis

We performed sensitivity analysis for all key variables to explore how results vary across plausible ranges. In univariable sensitivity analysis, all key variables were varied based on reported confidence intervals or with a variance of 20% from base-case values. We examined the uncertainty of the fitted PFS curves by expressing into median PFS. We performed 1,000 Monte Carlo simulations to conduct probabilistic sensitivity analysis, with the variables simultaneously varied with specified distributions. In the base-case, we assumed the proportion of patients receiving subsequent line therapy to be approximately 65% to 80%. Based on clinical observation and literature, more patients who had first-line docetaxel plus ADT or ADT alone would receive subsequent line therapy, since they would more likely be candidates for second-line targeted therapy, as compared to patients who had first-line abiraterone, enzalutamide, or apalutamide, whose second-line treatment would preferably be docetaxel. As an exploratory scenario analysis, we evaluated the impact of having a lower proportion of patients receiving subsequent line therapy after first-line docetaxel (i.e., 65%), similar to that for abiraterone, enzalutamide, and apalutamide plus ADT.

## Results

### Base-Case Results

Simulated results for the base-case are outlined in **Table 1**. Compared to treatment with ADT only, ADT plus docetaxel, abiraterone, and enzalutamide improved QALYs at higher costs incrementally. Compared with ADT alone, docetaxel plus ADT provided an additional 0.28 QALYs at a cost of US$12,870 per QALY. Compared with docetaxel plus ADT, abiraterone plus ADT provided an additional 1.70 QALYs at an ICER of US$38,897 for each QALY gained. Compared with abiraterone plus ADT, enzalutamide plus ADT gained an additional 0.87 QALYs at a cost of US$509,813 per QALY. Furthermore, apalutamide plus ADT was strongly dominated by enzalutamide plus ADT. Given the WTP threshold of US$100,000/QALY, abiraterone plus ADT represented high-value health care.

**Table 1:** Base-case results.

	Total			Incremental		ICER (US$/QALY)
Strategy	Cost (US$)	QALYs	Life-years	Cost (US$)	QALYs	
ADT only	69,554	3.42	4.34	—	—	Referent
DCX + ADT	73,144	3.70	4.69	3,590	0.28	12,870
AA + ADT	139,254	5.40	6.48	66,109	1.70	38,897
ENZ + ADT	583,783	6.27	7.47	444,529	0.87	509,813
APA + ADT	646,636	5.49	6.59	62,853	—	Dominated

QALY, quality-adjusted life-year; ICER, incremental cost-effectiveness ratio; DCX, docetaxel; ADT, androgen deprivation therapy; AA, abiraterone; APA, apalutamide; ENZ, enzalutamide.

### Sensitivity Analysis

**Figure 1:**
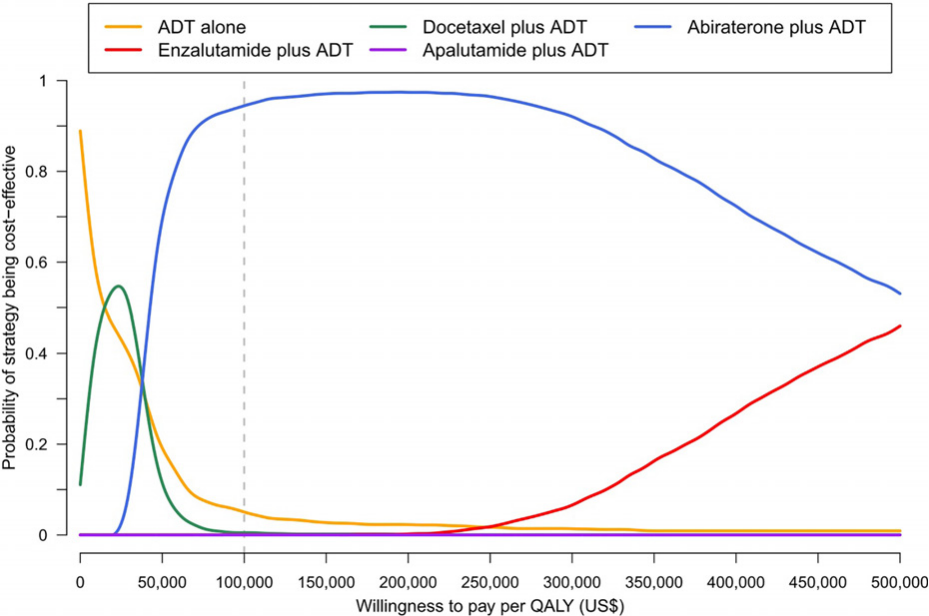
Cost-effectiveness acceptability curve.

Results of univariable sensitivity analysis showed that the variables with greatest influence on the ICER included drug costs of abiraterone and enzalutamide, median PFS of all strategies, and utilities of receiving abiraterone and enzalutamide (**Supplementary Table 4**). Abiraterone plus ADT remained the most preferred option across plausible ranges of parameters. The results of probabilistic sensitivity analyses suggested that abiraterone plus ADT is the strategy with the highest probability of being cost-effective at a WTP threshold of US$100,000 per QALY gained (**Figure 1**).

The results of the two-way sensitivity analysis demonstrated that abiraterone plus ADT remained the preferred option over docetaxel plus ADT (**Supplementary Figure 4**). At the base-case (i.e., median PFS 41.7 months) and worst-case (median PFS 34.1 months) efficacies, abiraterone plus ADT would be preferred when the monthly cost of abiraterone was below US$3,229 and US$2,710 respectively; both threshold costs were at least 1.8 times its current base-case cost (US$1,440). A separate two-way sensitivity analysis suggested that at the base-case, enzalutamide plus ADT would be a cost-effective alternative if enzalutamide cost drops to less than US$2,420 per month, i.e., 67% reduction from the current base-case cost (US$7,400) (**Supplementary Figure 5**). Even at its best estimated efficacy (median PFS 80.8 months), the monthly cost of enzalutamide has to drop by 58% to US$3,104 in order for enzalutamide to be recommended over abiraterone. In the scenario analysis, if the proportion of patients receiving subsequent line therapy after first-line docetaxel is lowered to 65%, the same as that for other combination treatment strategies, abiraterone was still more cost-effective compared to docetaxel, with an ICER of US$42,869 per QALY.

## Discussion

To our knowledge, this study is the first cost-effectiveness analysis of all approved drug options for mHSPC. Our analysis was based on US data, but we believe that our results can be applied to healthcare systems in other developed countries. Based on our results, abiraterone plus ADT is the preferred treatment at a WTP threshold of US$100,000 per QALY gained. The results from our study differs from a previously reported analysis (13), which suggested that docetaxel plus ADT was more cost-effective than abiraterone plus ADT, while the ICER of abiraterone plus ADT was US$295,212 per QALY gained compared with docetaxel plus ADT. Several factors may contribute to this difference.

First, with longer follow-up data of abiraterone from CHAARTED trial (5) and publication of a network meta-analysis (11) comparing across different treatment options, we were able to derive updated survival and toxicities data. Second, the drug cost of abiraterone was substantially reduced, to a greater extent than that of docetaxel. This is mainly attributed to the introduction of generic abiraterone, which costs less than one sixth of the brand name product. Moreover, drug costs were generally lower for the VA compared with private payers. Third, we took into account the differential costs of second-line treatment between treatment arms. From the literature, we observed that a lower proportion of patients who were given abiraterone, enzalutamide, or apalutamide would go on to receive second-line treatment, compared with patients given docetaxel plus ADT or ADT alone in the first line. In addition, adjusting for different patterns of second-line treatment, 14% of patients who received first-line docetaxel would be treated with cabazitaxel, which cost approximately US$12,000 per month, compared with 5% of patients given first-line abiraterone. This led to a relatively higher cost of second-line treatment for patients given docetaxel plus ADT and ADT alone. Nevertheless, from our scenario analysis, when a lower proportion of patients given first-line docetaxel received subsequent line therapy, abiraterone remained cost-effective compared to docetaxel.

There are several limitations to our analysis. First, outcomes data were derived from randomized controlled trials and a network meta-analysis. The patient selection criteria under these trial settings may limit the generalizability of our results in real-world clinical practice. Secondly, drug costs vary across health care systems and are expected to change over time. For instance, the introduction of generic abiraterone has evidently led to a lower incremental cost per QALY gained, making it a cost-effective treatment option. Moreover, drug costs in the model were derived from the VA, which are generally lower and better reflect the actual economic value of the drug, while prices paid by private payers and Medicare tend to be inflated. We performed sensitivity analyses to account for the wide variability in cost values. Thirdly, we did not include the use of radiotherapy in our comparison despite its proven role in low-volume disease. Fourthly, the follow-up duration of ENZAMET (8), ARCHES (9), and TITAN (10) trials were relatively short compared to trials for docetaxel and abiraterone. Longer follow-up data may change survival and toxicity outcomes for enzalutamide and apalutamide. Furthermore, we assumed that the probability from CRPC to death was the same across all strategies. Although model values were calibrated to published OS curves, the projected OS curves may be less well-fitted for some strategies (e.g., Docetaxel). Finally, we did not stratify mHSPC patients according to disease volume or risk as per CHAARTED (5) and LATITUDE (6) studies. Nevertheless, recent studies confirmed survival benefits of docetaxel and abiraterone regardless of volume and risk groups.

## Conclusions

From the US payer perspective, abiraterone plus ADT costs US$38,897 per QALY gained and is the preferred treatment option at a WTP threshold of US$100,000 per QALY gained in the first-line treatment of mHSPC.

## Data Availability Statement

The original contributions presented in the study are included in the article/**Supplementary Material**. Further inquiries can be directed to the corresponding author.

## Author Contributions

WS, TS, and HC conceived and designed the experiments. WS, HC, PL, and TS performed the experiments. WS, HC, PL, and TS analyzed the data. WS, TS, and HC wrote the manuscript. All authors contributed to the article and approved the submitted version.

## Conflict of Interest

The authors declare that the research was conducted in the absence of any commercial or financial relationships that could be construed as a potential conflict of interest.
