# Supplementary material for: A Cost-Effectiveness Analysis of Systemic Therapy for Metastatic Hormone-Sensitive Prostate Cancer
Source: Front Oncol. 2021 Feb 24;11:627083. doi: 10.3389/fonc.2021.627083 (PMC7943717; doi:10.3389/fonc.2021.627083)
Supplement: Supplementary file 1 [file DataSheet_1.pdf]

Supp Table 1: Model parameters: base-case values, ranges, and distributions for sensitivity analysis

Variable	Base-case value	Range		Distribution (parameters)	Reference for base-case value
		Minimum	Maximum		
Fitting to PFS curves (time-dependent)[a]:					
ADT alone				Lognormal(2.92, 1.20)	Calibrated
Docetaxel plus ADT				Lognormal(3.14, 1.14)	Calibrated
Abiraterone plus ADT				Lognormal(3.73, 1.34)	Calibrated
Enzalutamide plus ADT				Lognormal(4.08, 1.29)	Calibrated
Apalutamide plus ADT				Lognormal(3.80, 1.28)	Calibrated
Median PFS [b] (month):					
ADT alone	18.6	9.9	35.0		Projected [b]
Docetaxel plus ADT	23.1	20.9	25.5		Projected [b]
Abiraterone plus ADT	41.7	34.1	51.0		Projected [b]

Enzalutamide plus ADT	59.3	43.5	80.8		Projected [b]
Apalutamide plus ADT	44.6	38.8	51.4		Projected [b]
Monthly transition probability of prostate cancer death from CRPC	0.0415	0.0388	0.0449	Beta(608, 14,046)	Calibrated
Subsequent line therapy proportion					
ADT alone	80%	75%	85%	PERT(75%,80%,85%)	Assumption
Docetaxel	80%	75%	85%	PERT(75%,80%,85%)	Assumption
Abiraterone	65%	60%	70%	PERT(60%,65%,70%)	Assumption
Enzalutamide	65%	60%	70%	PERT(60%,65%,70%)	Assumption
Apalutamide	65%	60%	70%	PERT(60%,65%,70%)	Assumption
Monthly Utility					
HSPC treated with ADT	0.069	0.0595	0.0794	Beta(171.0, 2301.3)	[1]
In first-line Docetaxel	0.067	0.0602	0.0735	Beta(358.5, 5018.8)	[3]
In first-line Abiraterone	0.072	0.0632	0.0815	Beta(219.74, 2829.24)	[4]
In first-line Enzalutamide	0.072	0.0632	0.0815	Beta(219.74, 2829.24)	Assumption

In first-line Apalutamide	0.072	0.0632	0.0815	Beta(219.74, 2829.24)	Assumption
CRPC	0.061	0.0539	0.0677	Beta(277.46, 4299.54)	[5]
Monthly Drug Cost (US$)					
Eligard	55.62	44.72	66.52	Normal(55.62, 5.56)	[6]
Docetaxel	74.65	60.02	89.28	Normal(74.65, 74.7)	[6]
Abiraterone	1440.00	1157.77	1722.23	Normal(1440, 144)	[6]
Enzalutamide	7403.32	5952.30	8854.34	Normal(7403.32, 740.33)	[6]
Apalutamide	9823.04	7897.76	11748.32	Normal(9823.04, 982.30)	[6]
Cabazitaxel	12008.27	9654.69	14361.85	Normal(12008.27, 1200.83)	[6]
Mitoxantrone	145.25	116.78	173.72	Normal(145.25, 14.53)	[6]
Bicalutamide	657.30	528.47	786.13	Normal(657.3, 65.73)	[6]
Cost of chemotherapy administration (US$)	350.66	281.93	419.39	Normal(350.66, 35.07)	[7]
Cost of prostate cancer death (US$)	37184.14	29896.18	44472.10	Normal(37184.14, 3718.41)	[8]
Adverse event cost (US$)					

Febrile neutropenia	28,191	22665.67	33716.33	Normal(28191, 2819.1)	[9]
Neutropenia	18,965	15247.93	22682.07	Normal(18965,1896.5)	[10]
Infection with neutropenia	33,705	27098.94	40311.06	Normal(33705, 3370.5)	[11]
Diarrhea	18,224	14652.16	21795.84	Normal(18224, 1822.4)	[10]
Dyspnea	23,189	18644.04	27733.96	Normal(23189, 2318.9)	[10]
Fatigue	9,313	7487.69	11138.31	Normal(9313, 931.3)	[12]
Elevated ALT	5,709	4590.06	6827.94	Normal(5709, 570.9)	[13]
Elevated AST	5,709	4590.06	6827.94	Normal(5709, 570.9)	[13]
Rash	17,340	13941.42	20738.58	Normal(17340, 1734)	[10]
Hypertension	29,148	23435.10	34860.90	Normal(29148, 2914.8)	[10]
Hypokalemia	7,111	5717.27	8504.73	Normal(7111, 711.1)	[1]
Atrial fibrillation	27,856	22396.32	33315.68	Normal(27856, 2785.6)	[10]
Cardiac disorder	51,909	41735.02	62082.98	Normal(51909, 5190.9)	[14]
Syncope	8,253	6635.44	9870.56	Normal(8253, 825.3)	[15]
Fracture	4,714	3790.07	5637.93	Normal(4714, 471.4)	[16]
Adverse event disutility					

Febrile neutropenia	0.37	0.1833	0.5782	Beta(7.9, 13.5)	[17]
Neutropenia	0.09	0.0581	0.1278	Beta(23.0, 232.8)	[18]
Infection with neutropenia	0.4	0.2465	0.5644	Beta(14, 21)	[1]
Diarrhea	0.103	0.0627	0.1524	Beta(18, 156.3)	[19]
Dyspnea	0.1	0.0556	0.1549	Beta(13.7, 123.6)	[1]
Fatigue	0.115	0.0651	0.1757	Beta(14.31, 110.62)	[19]
Elevated ALT	0.05	0.0183	0.0974	Beta(5.7, 107.4)	[1]
Elevated AST	0.05	0.0183	0.0974	Beta(5.7, 107.4)	[1]
Rash	0.13	0.0734	0.1986	Beta(14.02, 94.23)	[20]
Hypertension	0	0	0	-	[19]
Hypokalemia	0.04	0.0189	0.0685	Beta(9.4, 225.6)	[1]
Atrial fibrillation	0.164	0.1138	0.2216	Beta(29.5, 150.2)	[21]
Cardiac disorder	0.33	0.1784	0.5002	Beta(10.26, 20.93)	[22]
Syncope	0.2	0.1114	0.3050	Beta(12.66, 50.89)	Assumption
Fracture	0.15	0.0844	0.2290	Beta(13.63, 77.58)	[23]
Docetaxel AE incidence					

Febrile neutropenia	0.0173	0.00793	0.02418	Beta(12.66, 832.82)	[24, 25, 26]
Neutropenia	0.0243	0.01278	0.04743	Beta(9.18, 325.23)	[24, 25, 26]
Infection with neutropenia	0.0020	0.00095	0.00551	Beta(5.41, 1958.04)	[24, 25, 26]
Diarrhea	0.0070	0.00051	0.00803	Beta(2.47, 789.43)	[24, 25, 26]
Dyspnea	0.0047	0.00094	0.00752	Beta(4.02, 1163.20)	[24, 25, 26]
Fatigue	0.0041	0.00181	0.01125	Beta(5.04, 908.49)	[24, 25, 26]
Abiraterone AE incidence					
Elevated ALT	0.0023	0.00188	0.00282	Beta(92.17, 39553.03)	[27, 28]
Elevated AST	0.0010	0.00022	0.00209	Beta(3.53, 3843.81)	[27, 28]
Hypertension	0.0017	0.00124	0.00227	Beta(41.67, 24242.44)	[27, 28]
Hypokalemia	0.0020	0.00019	0.00437	Beta(1.98, 1262.49)	[27, 28]
Atrial fibrillation	0.0004	0.00008	0.00078	Beta(3.44, 10162.2)	[27, 28]
Cardiac disorder	0.0008	0.00025	0.00167	Beta(4.78, 5949.32)	[27, 28]
Enzalutamide AE incidence					
Neutropenia	0.0009	0.00003	0.00143	Beta(1.43, 3175.63)	[29, 30]
Fatigue	0.0011	0.00030	0.00196	Beta(4.77, 5055.59)	[29, 30]

Hypertension	0.0016	0.00064	0.00276	Beta(7.62, 5030.42)	[29, 30]
Syncope	0.0007	0.00018	0.00127	Beta(4.42, 7407.88)	[29, 30]
Apalutamide AE incidence					
Rash	0.0028	0.00191	0.00380	Beta(32.65, 11728.29)	[31]
Fracture	0.0006	0.00023	0.00112	Beta(6.51, 11042.69)	[31]
Fatigue	0.0007	0.00028	0.00124	Beta(7.45, 11057.75)	[31]

[a] The monthly transition probabilities from progression free to CRPC were time-dependent based on the lognormal distribution (μ (location), σ (scale)), estimated from fitting to the published PFS curves.

[b] The median PFS values were projected from the fitted lognormal distribution.

References:

[1] Sathianathen NJ, Alarid-Escudero F, Kuntz KM, et al. A Cost-effectiveness Analysis of Systemic Therapy for Metastatic Hormone-sensitive Prostate Cancer. European Urology Oncology. 2019;2(6):649-655. doi:10.1016/j.euo.2019.01.004

[2] Sathianathen NJ, Koschel S, Thangasamy IA, et al. Indirect Comparisons of Efficacy between Combination Approaches in Metastatic Hormone-sensitive Prostate Cancer: A Systematic Review and Network Meta-analysis. European Urology. 2020;77(3):365-372. doi:10.1016/j.eururo.2019.09.004

[3] Morgans AK, Chen Y-H, Sweeney CJ, et al. Quality of Life During Treatment With Chemohormonal Therapy: Analysis of E3805 Chemohormonal Androgen Ablation Randomized Trial in Prostate Cancer. JCO. 2018;36(11):1088-1095. doi:10.1200/JCO.2017.75.3335

[4] Feyerabend S, Saad F, Li T, et al. Survival benefit, disease progression and quality-of-life outcomes of abiraterone acetate plus prednisone versus docetaxel in metastatic hormone-sensitive prostate cancer: A network meta-analysis. European Journal of Cancer. 2018;103:78-87. doi:10.1016/j.ejca.2018.08.010

[5] Lloyd AJ, Kerr C, Penton J, Knerer G. Health-Related Quality of Life and Health Utilities in Metastatic Castrate-Resistant Prostate Cancer: A Survey Capturing Experiences from a Diverse Sample of UK Patients. Value in Health. 2015;18(8):1152-1157. doi:10.1016/j.jval.2015.08.012

[6] U.S. Department of Veterans Affairs. National Acquisition Center Contract Catalog Search Tool. https://www.vendorportal.ecms.va.gov/NAC/Pharma/List. Accessed July 4, 2020.

[7] Kruse GB, Amonkar MM, Smith G, Skonieczny DC, Stavrakas S. Analysis of costs associated with administration of intravenous single-drug therapies in metastatic breast cancer in a U.S. population. J Manage Care Pharm 2008;14:844–57.

[8] Chastek B, Harley C, Kallich J, Newcomer L, Paoli CJ, Teitelbaum AH. Health care costs for patients with cancer at the end of life. J Oncol Pract 2012;8:75–80.

[9] Tai E, Guy GP, Dunbar A, Richardson LC. Cost of Cancer-Related Neutropenia or Fever Hospitalizations, United States, 2012. Journal of Oncology Practice. 2017;13:e552-e61.

[10] Wong W, Yim YM, Kim A, et al. Assessment of costs associated with adverse events in patients with cancer. PLOS ONE. 2018;13(4):e0196007. doi:10.1371/journal.pone.0196007

[11] Schilling MB, Parks C, Deeter RG. Costs and outcomes associated with hospitalized cancer patients with neutropenic complications: A retrospective study. Experimental and therapeutic medicine. 2011;2:859-66.

[12] Roy A, Kish JK, Bloudek L, et al. Estimating the Costs of Therapy in Patients with Relapsed and/or Refractory Multiple Myeloma: A Model Framework. Am Health Drug Benefits. 2015;8(4):204-215.

[13] Rashid N, Koh HA, Baca HC, Lin KJ, Malecha SE, Masaquel A. Economic burden related to chemotherapy-related adverse events in patients with metastatic breast cancer in an integrated health care system. Breast cancer (Dove Medical Press). 2016;8:173-81.

[14] Bilir SP, Ma Q, Zhao Z, Wehler E, Munakata J, Barber B. Economic Burden of Toxicities Associated with Treating Metastatic Melanoma in the United States. Am Health Drug Benefits. 2016;9(4):203-213.

[15] Sun BC, Emond JA, Camargo CA. Direct medical costs of syncope-related hospitalizations in the United States. The American Journal of Cardiology. 2005;95(5):668-671.

[16] Institute for Clinical and Economic Review, 2018 Model Analysis Plan: Antiandrogen therapies for non-metastatic castration-resistant prostate cancer

[17] Fust K, Li X, Maschio M, Villa G, Parthan A, Barron R, et al. Cost-Effectiveness Analysis of Prophylaxis Treatment Strategies to Reduce the Incidence of Febrile Neutropenia in Patients with Early-Stage Breast Cancer or Non-Hodgkin Lymphoma. PharmacoEconomics. 2017;35:425-38.

[18] Hornberger J, Hirsch FR, Li Q, Page RD. Outcome and economic implications of proteomic test-guided second- or third-line treatment for advanced non-small cell lung cancer: Extended analysis of the PROSE trial. Lung Cancer. 2015;88:223-30.

[19] Shlomai A, Leshno M, Goldstein DA. Regorafenib treatment for patients with hepatocellular carcinoma who progressed on sorafenib—A cost-effectiveness analysis. Kim DY, ed. PLoS ONE. 2018;13(11):e0207132. doi:10.1371/journal.pone.0207132

[20] Matza LS, Sapra SJ, Dillon JF, et al. Health state utilities associated with attributes of treatments for hepatitis C. Eur J Health Econ. 2015;16(9):1005-1018. doi:10.1007/s10198-014-0649-6

[21] Vilain KA, Yang MC, Hui Tan EC, Wang K, Li H, Hsu WH, et al. Cost-Effectiveness of Edoxaban vs. Warfarin in Patients with Atrial Fibrillation Based on Results of the ENGAGE AF - TIMI 48 Trial: Taiwanese Perspective. Value in health regional issues. 2017;12:74-83.

[22] Matza LS, Stewart KD, Gandra SR, et al. Acute and chronic impact of cardiovascular events on health state utilities. *BMC Health Serv Res*. 2015;15. doi:10.1186/s12913-015-0772-9

[23] Lobo JM, Trifiletti DM, Sturz VN, et al. Cost-effectiveness of the Decipher Genomic Classifier to Guide Individualized Decisions for Early Radiation Therapy After Prostatectomy for Prostate Cancer. Clinical Genitourinary Cancer. 2017;15(3):e299-e309. doi:10.1016/j.clgc.2016.08.012

[24] Gravis G, Fizazi K, Joly F, et al. Androgen-deprivation therapy alone or with docetaxel in non-castrate metastatic prostate cancer (GETUG-AFU 15): a randomised, open-label, phase 3 trial. *The Lancet Oncology*. 2013;14(2):149-158. doi:10.1016/S1470-2045(12)70560-0

[25] James ND, Sydes MR, Clarke NW, et al. Addition of docetaxel, zoledronic acid, or both to first-line long-term hormone therapy in prostate cancer (STAMPEDE): survival results from an adaptive, multiarm, multistage, platform randomised controlled trial. *The Lancet*. 2016;387(10024):1163-1177. doi:10.1016/S0140-6736(15)01037-5

[26] Sweeney CJ, Carducci M, Liu G, et al. Chemohormonal Therapy in Metastatic Hormone-Sensitive Prostate Cancer. *New England Journal of Medicine*. 2015;373(8):737-746. doi:10.1056/NEJMoa1503747

[27] James ND, de Bono JS, Spears MR, et al. Abiraterone for Prostate Cancer Not Previously Treated with Hormone Therapy. *New England Journal of Medicine*. 2017;377(4):338-351. doi:10.1056/NEJMoa1702900

[28] Fizazi K, Tran N, Fein L, et al. Abiraterone plus Prednisone in Metastatic, Castration-Sensitive Prostate Cancer. *New England Journal of Medicine*. 2017;377(4):352-360. doi:10.1056/NEJMoa1704174

[29] Armstrong AJ, Szmulewitz RZ, Petrylak DP, et al. ARCHES: A Randomized, Phase III Study of Androgen Deprivation Therapy With Enzalutamide or Placebo in Men With Metastatic Hormone-Sensitive Prostate Cancer. *JCO*. 2019;37(32):2974-2986. doi:10.1200/JCO.19.00799

[30] Davis ID, Martin AJ, Stockler MR, et al. Enzalutamide with Standard First-Line Therapy in Metastatic Prostate Cancer. *New England Journal of Medicine*. Published online June 2, 2019. doi:10.1056/NEJMoa1903835

[31] Chi KN, Agarwal N, Bjartell A, et al. Apalutamide for Metastatic, Castration-Sensitive Prostate Cancer. *N Engl J Med*. 2019;381(1):13-24. doi:10.1056/NEJMoa1903307

Supp Table 2: Background mortality rate

Reference: Arias E et al. United States Life Tables, 2017. National Vital Statistics Reports. 2019; Vol. 68, No. 7, page 1-66

Age	Background mortality rate	Age	Background mortality rate
60	0.01147	81	0.062514
61	0.012361	82	0.069452
62	0.01326	83	0.077622
63	0.01414	84	0.086155
64	0.015019	85	0.09545
65	0.015942	86	0.105788
66	0.017026	87	0.118527
67	0.018189	88	0.132437
68	0.019483	89	0.147541
69	0.02099	90	0.163839
70	0.022448	91	0.181308
71	0.024631	92	0.1999
72	0.02657	93	0.219535
73	0.02904	94	0.240108
74	0.031539	95	0.26148
75	0.034644	96	0.283491

76	0.038148	97	0.305955
77	0.04225	98	0.328673
78	0.046522	99	0.351434
79	0.051401	100	1
80	0.056783		

Supp Table 3: Drug dose and costs

Drug	Dose	Unit price ($)	Cost for 1 model cycle ($/month)
Eligard	22..5mg every 3 months	166.86/22.5mg	55.62
Docetaxel	75mg/m2 (70kg, 175cm) every 3 weeks	55.99/160mg	74.65
Abiraterone	1000mg/day	1,440 for 250mg x 120tab	1,440.00
Enzalutamide	160mg/day	7,403.32 for 40mg x 120tab	7,403.32
Apalutamide	240mg/day	9,823.04 for 60mg x 120tab	9,823.04
Cabazitaxel	20mg/m2 (70kg, 175cm) every 3 weeks	9,006.2/60mg	12,008.27
Mitoxantrone	10mg/m2 (70kg, 175cm) every 3 weeks	108.94/20mg	145.25
Bicalutamide	50mg/day	657.3 for 50mg x 30tab	657.30

Supp Table 4: Results of Univariable Sensitivity Analysis

**Model parameter** [a]	**Base case value (95% CI)**	**ICER** [b] **($/QALY)**	
		At parameter's lower limit	At parameter's upper limit
Median PFS of ADT alone (months)	18.6 (9.9-35.0)		
Docetaxel		560	Strongly dominated
Abiraterone		38,897	100,284
Enzalutamide		509,813	509,813
Median PFS of Docetaxel plus ADT (months)	23.1 (20.9-25.5)		
Docetaxel		Weakly dominated (40,836)	6,146
Abiraterone		35,227	44,141
Enzalutamide		509,813	509,813
Median PFS of Abiraterone plus ADT (months)	41.7 (34.1-51.0)		
Docetaxel		12,870	12,870

Abiraterone		47,048	34,111
Enzalutamide		334,748	1,211,221
Median PFS of Enzalutamide plus ADT (months)	59.3 (43.5-80.8)		
Docetaxel		12,870	12,870
Abiraterone		38,897	38,897
Enzalutamide [a]		Weakly dominated (7,815,956)	308,196
Monthly transition probability of prostate cancer death from CRPC	0.0415 (0.0388-0.0449)		
Docetaxel		12,158	13,643
Abiraterone		38,896	38,996
Enzalutamide		512,393	507,015
Cost of eligard	55.62 (44.72-66.52)		
Docetaxel		12,705	13,035
Abiraterone		38,759	39,035
Enzalutamide		509,664	509,961
Cost of docetaxel	74.65 (60.02-89.28)		
Docetaxel		12,623	13,117

Abiraterone		38,930	38,863
Enzalutamide		509,815	509,810
Cost of abiraterone	1,440 (1157.77-1722.23)		
Docetaxel		13,060	12,679
Abiraterone		29,255	48,538
Enzalutamide		528,798	490,827
Cost of enzalutamide	7,403.32 (5952.3-8854.34)		
Docetaxel		15,445	10,294
Abiraterone		39,254	38,540
Enzalutamide		390,494	629,131
Cost of cabazitaxel	12,008.27 (9654.69-14361.85)		
Docetaxel		9,508	16,231
Abiraterone		39,668	38,126
Enzalutamide		509,858	509,767
Cost of mitoxantrone	145.25 (116.78-173.72)		
Docetaxel		12,866	12,873
Abiraterone		38,904	38,890
Enzalutamide		509,813	509,813
Cost of bicalutamide	657.3 (528.47-786.13)		
Docetaxel		12,448	13,292

Abiraterone		39,201	38,593
Enzalutamide		509,813	509,813
Cost of prostate cancer death	37184.14 (29896.18-44472.1)		
Docetaxel		13,306	12,434
Abiraterone		39,350	38,444
Enzalutamide		510,301	509,324
ADT utility per month	0.069 (0.0595-0.0794)		
Docetaxel		12,582	13,192
Abiraterone		33,128	47,794
Enzalutamide		509,813	509,813
Docetaxel utility per month	0.067 (0.0602-0.0735)		
Docetaxel		14,666	11,396
Abiraterone		38,130	39,740
Enzalutamide		509,813	509,813
Abiraterone utility per month	0.072 (0.0632-0.0815)		
Docetaxel		12,870	12,870
Abiraterone		56,609	29,188
Enzalutamide		316,679	1,449,975

Enzalutamide utility per month	0.072 (0.0632-0.0815)		
Docetaxel		12,870	12,870
Abiraterone		38,897	38,897
Enzalutamide		1,998,970	284,490
CRPC utility per month	0.061 (0.0539-0.0677)		
Docetaxel		25,245	8,488
Abiraterone		36,000	42,498
Enzalutamide		509,813	509,813

[a] Apalutamide was strongly dominated in all scenarios except when the median PFS for enzalutamide plus ADT was at its lower limit at 43.5 months (ICER was $7,815,956 for enzalutamide plus ADT vs abiraterone plus ADT and $5,320,670 for apalutamide plus ADT vs abiraterone plus ADT).

[b] The ICERs represent comparisons of (i) Docetaxel plus ADT vs ADT alone; (ii) Abiraterone plus ADT vs Docetaxel plus ADT; and (iii) Enzalutamide plus ADT vs Abiraterone plus ADT.

Supp Figure 1: State Transition Diagram

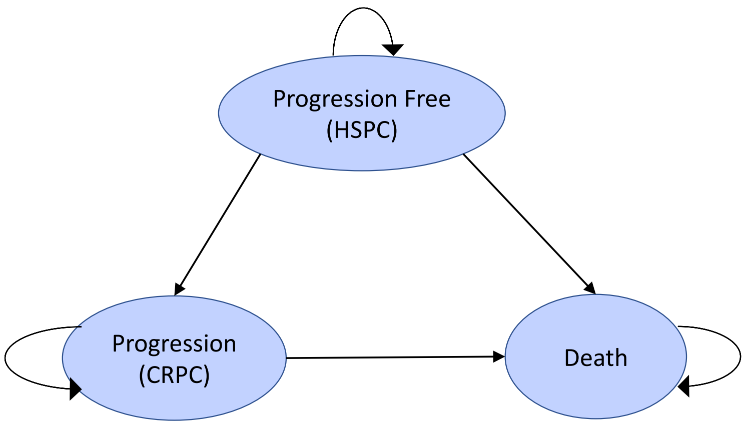

Supp Figure 2a: Decision tree

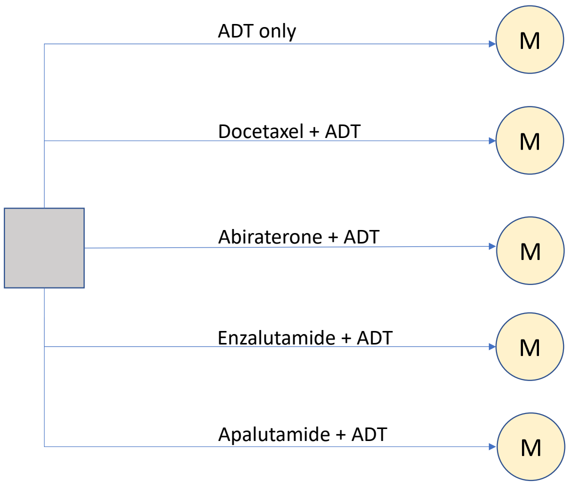

Supp Figure 2b: Markov Model

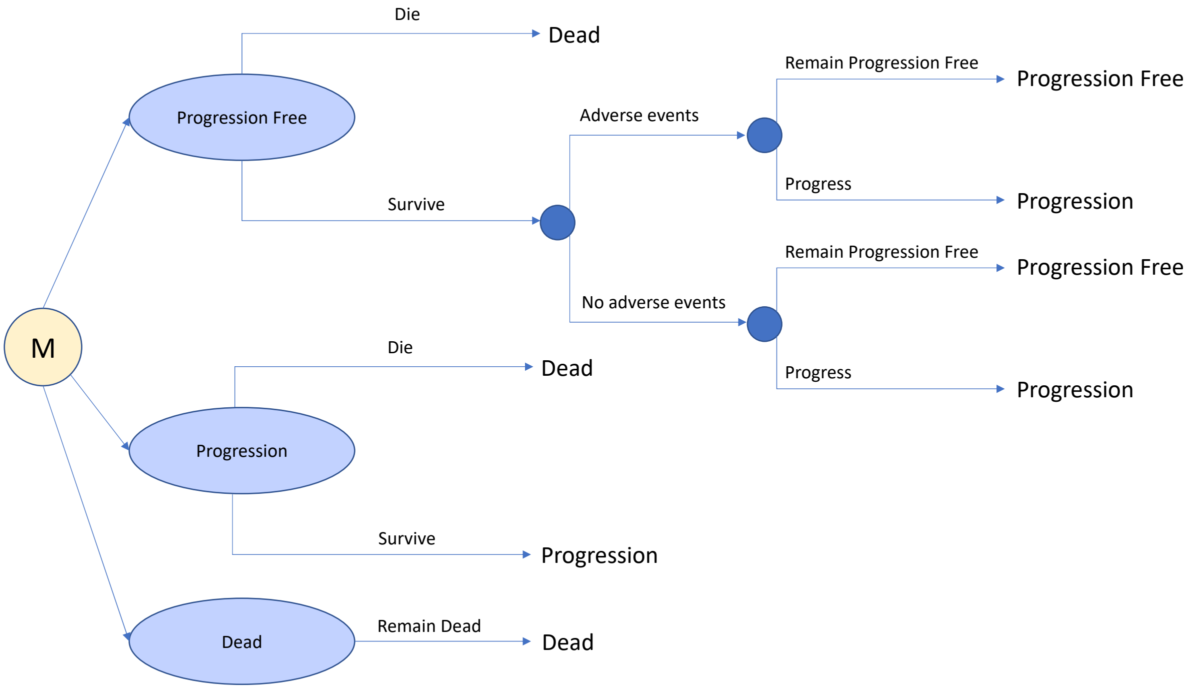

Supp Figure 3. Fitting of progression-free survival (PFS) and overall survival (OS) curves

PFS curves were fitted to log-normal distribution which was chosen based on Akaike information criterion. To estimate the monthly transition probability from CRPC to death, we first calculated the difference between the projected and published OS curves for each strategy. The best-fit transition probability from CPRC to death was chosen for generating the smallest overall sum of the squared difference among all strategies. The uncertainty of this probability was obtained by bootstrapping using 1,000 resampling.

The grey dashed lines represent the best-fitted lognormal projection; and the grey regions represent the confidence band of the fitting.

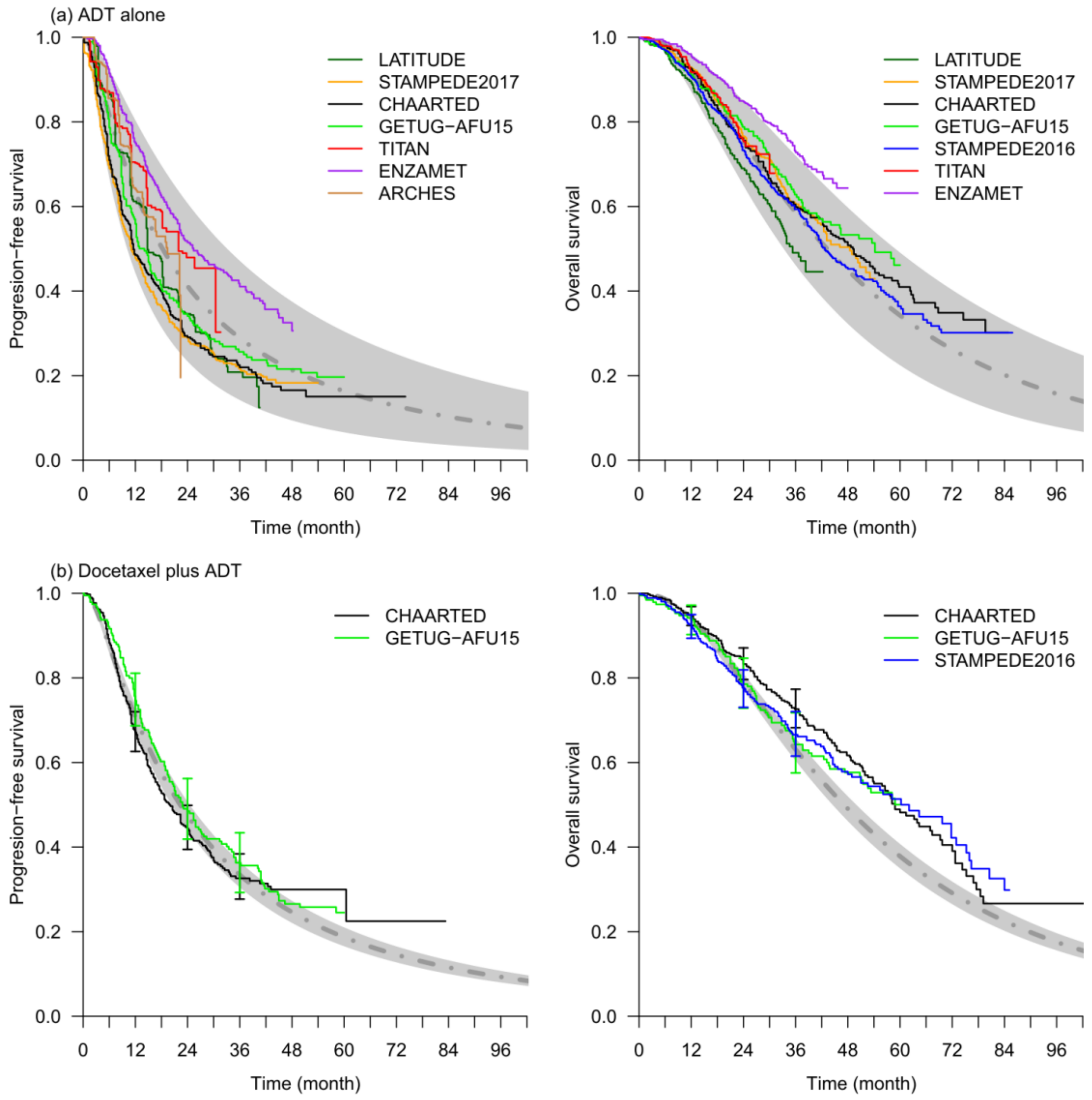

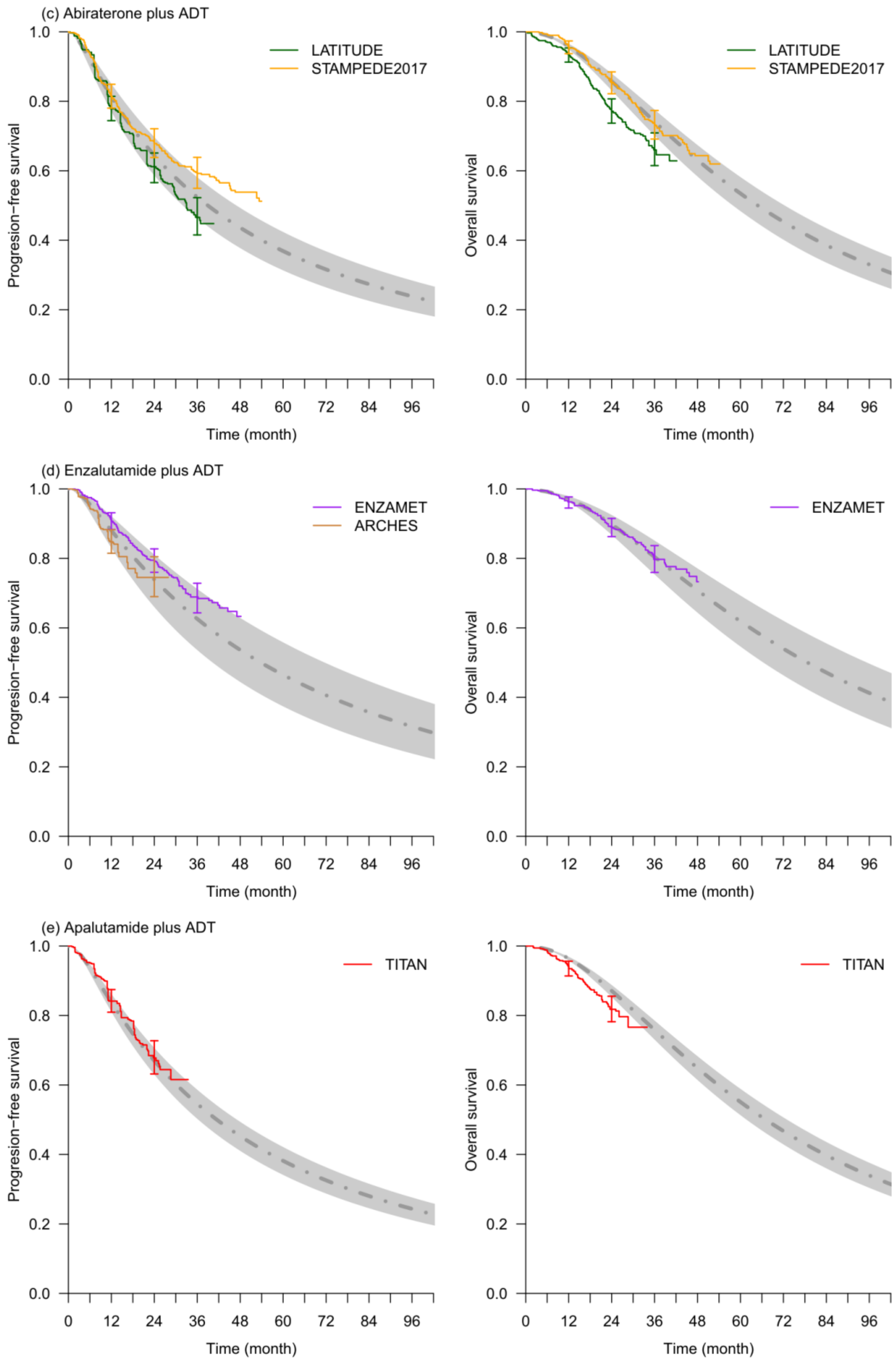
(c) Abiraterone plus ADT
LATITUDE
STAMPEDE2017
Progresion−free survival
Overall survival
Time (month)
(d) Enzalutamide plus ADT
ENZAMET
ARCHES
Progresion−free survival
Overall survival
Time (month)
(e) Apalutamide plus ADT
TITAN
Progresion−free survival
Overall survival
Time (month)

Supp Figure 4. Two-way sensitivity analysis of abiraterone plus ADT vs docetaxel plus ADT on median PFS and cost of abiraterone

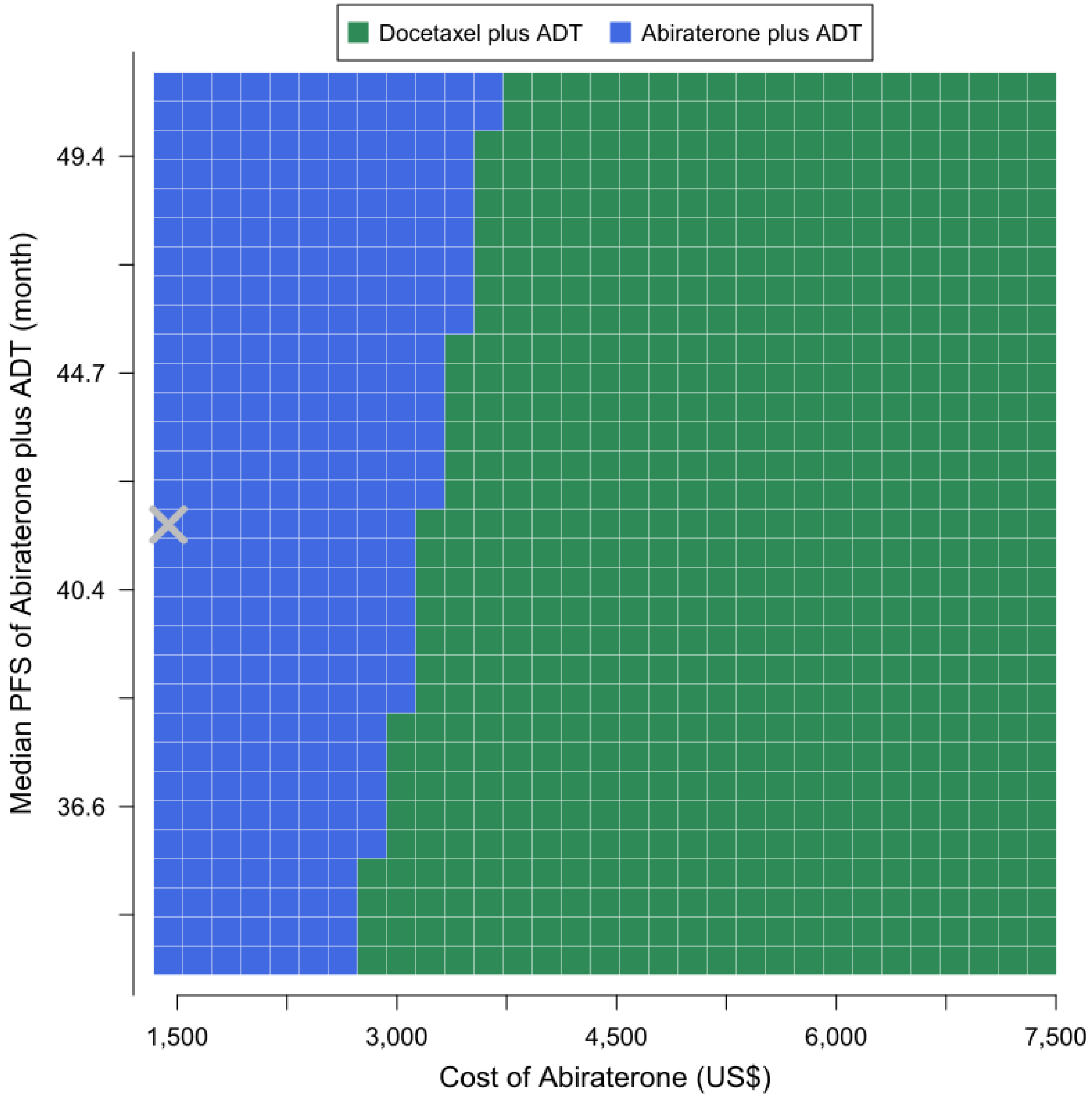

[a] The 3-year PFS probabilities were derived from the fitted log-normal distribution and therefore were not linearly spaced

[b] The grey cross denotes the cost and median PFS at base-case

Supp Figure 5. Two-way sensitivity analysis of enzalutamide plus ADT vs abiraterone plus ADT on median PFS and cost of enzalutamide

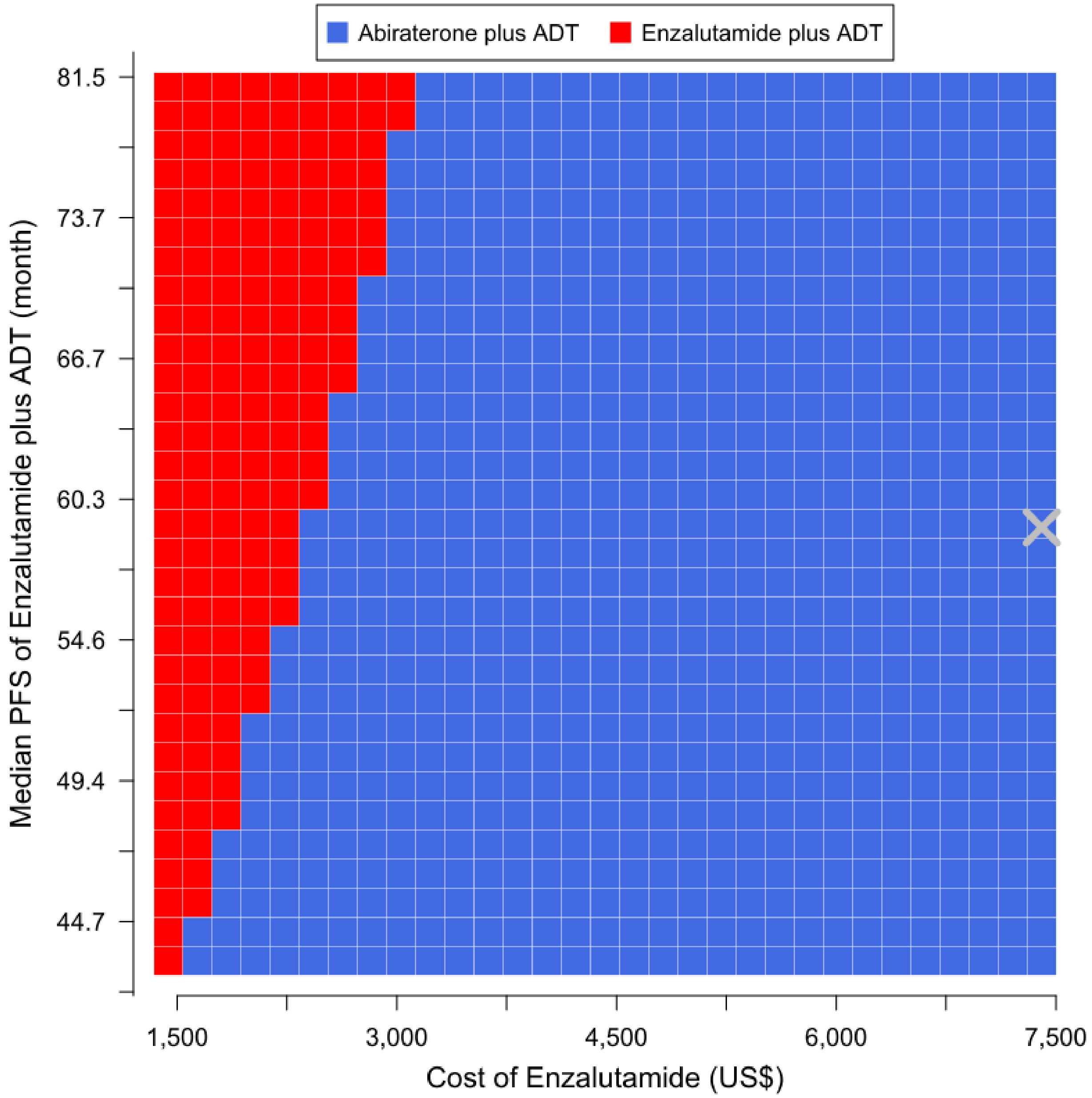

[a] The median PFS were derived from the fitted log-normal distribution and therefore were not linearly spaced

[b] The grey cross denotes the cost and median PFS at base-case
